# Investigating the Parasitoid Community Associated with the Invasive Mealybug *Phenacoccus solenopsis* in Southern China

**DOI:** 10.3390/insects12040290

**Published:** 2021-03-26

**Authors:** Hua-Yan Chen, Hong-Liang Li, Hong Pang, Chao-Dong Zhu, Yan-Zhou Zhang

**Affiliations:** 1 Key Laboratory of Zoological Systematics and Evolution, Institute of Zoology, Chinese Academy of Sciences, Beijing 100101, China; chenhuayan@mail.sysu.edu.cn (H.-Y.C.); lihongliang_2019@163.com (H.-L.L.); zhucd@ioz.ac.cn (C.-D.Z.); 2 State Key Laboratory of Biocontrol, Ecology and Evolution, School of Life Sciences, Sun Yat-sen University, Guangzhou 510275, China; Lsshpang@mail.sysu.edu.cn

**Keywords:** biological control, DNA barcoding, integrated taxonomy, species delimitation

## Abstract

**Simple Summary:**

The cotton mealybug *Phenacoccus solenopsis* Tinsley (Hemiptera: Pseudococcidae) is an emerging invasive insect pest in China. Hymenopteran parasitoids have great potential to be used as biological agents to suppress the populations of *P. solenopsis* in the field. We used an integrated approach of species delimitation, combining morphology with molecular data, to investigate the parasitoid community associated with *P. solenopsis* from south China. On the basis of both morphological and molecular evidence, we identified 18 hymenopteran parasitoid species belonging to 11 genera of four families. Among these species, eight species are primary parasitoids with *Aenasius arizonensis* (Girault) (Hymenoptera: Encyrtidae) being the dominant taxon, while the other 10 species are probably hyperparasitoids. These findings can assist in the utility of these parasitoids in the biological control of *P. solenopsis*.

**Abstract:**

The cotton mealybug *Phenacoccus solenopsis* Tinsley (Hemiptera: Pseudococcidae), is an emerging invasive insect pest in China. Hymenopteran parasitoids are the key organisms for suppressing populations of *P. solenopsis* in the field, and therefore could be used as biological agents. Accurate identification of the associated parasitoids is the critical step to assess their potential role in biological control. In this study, we facilitated the identification of the parasitoid composition of *P. solenopsis* using an integrated approach of species delimitation, combining morphology with molecular data. Eighteen Hymenoptera parasitoid species belonging to 11 genera of four families are recognized based on morphological examination and molecular species delimitation of the mitochondrial cytochrome c oxidase 1 (*COI*) gene and the 28S rDNA using the automatic barcode gap discovery (ABGD) and the Bayesian Poisson tree processes model (bPTP). Among these species, eight species are primary parasitoids with *Aenasius arizonensis* (Girault) (Hymenoptera: Encyrtidae) being the dominant taxon, while the other 10 species are probably hyperparasitoids, with a prevalence of *Cheiloneurus nankingensis* Li & Xu (Hymenoptera: Encyrtidae). These results indicate that parasitoid wasps associated with *P. solenopsis* from China are diverse and the integrated taxonomic approach applied in this study could enhance the accurate identification of these parasitoids that should be assessed in future biological control programs.

## 1. Introduction

The mealybug *Phenacoccus solenopsis* Tinsley (Hemiptera: Pseudococcidae), which originating from the USA, has become an emerging invasive and polyphagous pest in Asia [1,2,3,4,5,6]. It has been reported to feed on at least 213 host plant species belonging to 56 families [7], including the economically important cotton [5,6,8]. This mealybug species has caused serious yield losses of cotton in some Asian countries, particularly India and Pakistan [9,10]. Since its first report in mainland China in 2008, this mealybug has spread to 16 provinces of the country by the end of 2018 [11]. It was estimated that all cotton-growing regions of China could be infested by this mealybug [5].

Currently, chemical control is still the main strategy against *P. solenopsis* worldwide. However, due to the presence of a waxy coating over the body, ovoviviparous mode of reproduction (parthenogenesis with ovoviviparity is dominant over the oviparous mode), and overlapping generations and their cryptic habits, effective management of this pest is often limited to conventional insecticides [12]. In addition, repeated and overuse of insecticides have resulted in resistance and tolerance of *P. solenopsis* to some insecticides, and a negative impact on natural enemies, which could cause the resurgence of this pest [13,14,15,16]. Therefore, strategies for sustainable management of *P. solenopsis*, including biological control, have attracted increasing attention in recent years. Biological control involving natural enemies, especially parasitoids, has been thought to be the key factor in suppressing the populations of *P. solenopsis* in nature [12]. At least 38 species of parasitoids or hyperparasitoids (Table 1) belonging to six hymenopteran families have been reported to be associated with *P. solenopsis* throughout its distribution range [17,18,19,20,21,22,23,24,25,26,27,28,29]. Further assessment and application of these parasitoid wasps in biological control programs against *P. solenopsis* require an accurate identification of the species [28]. However, given the diverse guild of wasps associated with *P. solenopsis*, species identification based on morphology is not easy to handle, particularly for practitioners who are not the specialists of parasitoids, because these parasitoids are usually small and morphologically similar and require extensive examination of detailed morphological characters. DNA barcoding (the partial sequencing of the gene cytochrome c oxidase 1, *COI*) has become a sufficient species identification tool for insects [30,31]. Studies have shown that DNA barcoding is a useful tool for the identification of various groups of parasitoids [32,33,34,35]. In this study, we use an integrated approach (morphology combined with DNA barcoding) to identify the parasitoids associated with *P. solenopsis* from China. The results from this study may help to improve the determination of parasitoid species of *P. solenopsis*, which is a key step toward using parasitoids as biological control agents against this serious pest.

## 2. Materials and Methods

### 2.1. Parasitoid Collecting and Sampling

During 2010–2017, we surveyed the parasitoids of *P. solenopsis* in 9 provinces of southern China (Hainan, Guangdong, Guangxi, Jiangxi, Yunnan, Zhejiang, Fujian, Hunan and Hubei provinces). Branches and leaves of various host plants infested by *P. solenopsis* were collected from the field and brought back to the Key Laboratory of Zoological Systematics and Evolution, Institute of Zoology, Chinese Academy of Sciences (IZCAS), and kept in the greenhouse and checked daily for parasitoid emergence. Any parasitoids emerged were collected, preserved in 100% ethanol, and stored at −20 °C until further morphological and molecular studies. All specimens were identified to genus and provisional morphospecies. At least two specimens of each morphospecies from each locality were selected for DNA sequencing. After DNA extraction, the specimens were re-examined to confirm their morphological identification. All specimens (including DNA vouchers, Table S1) in this study are deposited at the Institute of Zoology, Chinese Academy of Sciences (IZCAS).

#### DNA Extraction, Amplification, and Sequencing

Overall, 212 representative specimens of 18 morphospecies were used for DNA barcoding analysis (see Table S2). Genomic DNA was extracted from each specimen using the DNeasy Blood & Tissue Kit (Qiagen, Hilden, Germany), following the manufacturer’s protocols. Polymerase chain reaction (PCR) amplification of two DNA fragments, mitochondrial DNA (mtDNA) cytochrome c oxidase 1 (*COI*) and nuclear 28S rRNA D1–2 (*28S*), were carried out on an Eppendorf thermal cycler and performed in 50 μL volumes containing 5 μL DNA template, 24.5 μL ddH_2_O, 5 μL 10× buffer, 5 μL MgCl_2_, 8 μL dNTP, 1 μL of each primer, and 0.5 μL LaTaq (Takara, Shiga, Japan). Primers [36,37,38,39,40,41], fragment length, and references are shown in Table S3. The PCR programs followed Chesters et al. [42]. The PCR products were visualized by 1% agarose gel electrophoresis. Products were purified and sequenced in both directions using BigDye v3.1 on an ABI 3730xl DNA analyzer (Applied Biosystems, Foster City, CA, USA). Chromatograms were assembled with Sequencing Analysis 6 (ThermoFisher Scientific, Gloucester, UK). All the amplified sequences were deposited in GenBank (accession numbers in Table S2).

### 2.2. Sequence Analysis and Molecular Species Delimitation

All sequences were blasted in the BOLD (Barcode of Life Database, http://www.barcodinglife.org/index.php/IDS_OpenIdEngine) and GenBank. The blast hits with over 97% similarity were recorded. Sequences were aligned using MAFFT v7.470 by the Q-INS-I strategy for 28S and G-INS-I strategy for *COI* [43]. The two fragments were concatenated in Geneious 11.0.3. In the final 1302 bp concatenated alignment, 28S and *COI* were 672 bp and 630 bp, respectively. Genetic Kimura-2 parameter (K2P) distances within and between species were calculated in MEGA 7 with pairwise deletion for gaps [44].

Distance and evolutionary model-based methods were both tested for molecular species delimitation. The automatic barcode gap discovery (ABGD) is a distance-based method that sorts the sequences into hypothetical species by partitioning and comparing the difference between sequences to identify a “barcode gap” [45]. The ABGD analyses were performed with the COI dataset on the web interface (http://wwwabi.snv.jussieu.fr/public/abgd/), using the default priors, Pmin = 0.001, Pmax = 0.1, Steps 10, and with barcode relative gap width = 1.00. The Poisson tree processes model (PTP) tests species boundaries on non-ultrametric phylogenetic trees by detecting significant differences in the number of substitutions between species and within species [46]; Bayesian Poisson tree processes model (bPTP) is an updated version of the original PTP with Bayesian posterior probability, providing more accurate results. For bPTP analyses, after removing the identical sequences, a maximum likelihood (ML) tree was generated in RAxML v8.2.10 under the GTRGAMMA evolutionary model and performed on the bPTP web server (https://species.h-its.org/ptp/), with default parameters. The COI and 28S sequences of *Hybrizon buccatus* (de Brebisson) (Hymenoptera: Ichneumonidae, GenBank: KU753286 (COI), KU753494 (28S)) were selected as the outgroup because of the close relationship between Ichneumonoidea and Chalcidoidea + Platygastroidea [47].

## 3. Results and Discussion

**Figure 1:**
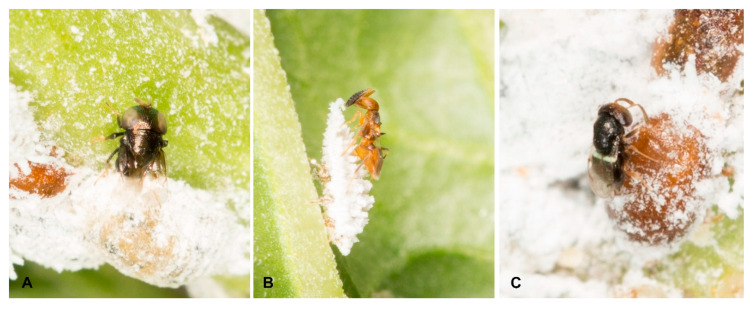
(**A**) Aenasius arizonensis parasitizing Phenacoccus solenopsis; (**B**) *Cheiloneurus nankingensis* parasitizing Phenacoccus solenopsis via probably Aenasius arizonensis; (**C**) Promuscidea unfasciativentris parasitizing a mummy of Phenacoccus solenopsis.

**Figure 2:**
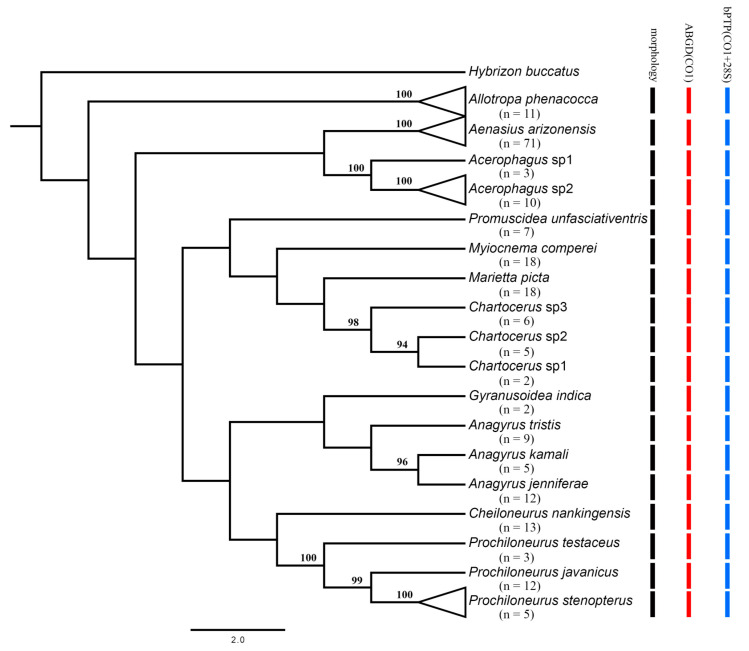
Maximum likelihood tree and results of species delimitation, only values > 90 for bootstrap are labeled.

A total of 870 individual parasitoid wasps emerged from 62 samples of *P. solenopsis* infecting 11 plant species from nine provinces of southern China (Table S2). Of these specimens, the initial morphological identifications suggested a total of 18 morphospecies belonging to four hymenopteran families as follows: Aphelinidae (3): *Marietta picta* (André), *Myiocnema comperei* Ashmead, *Promuscidea unfasciativentris* Girault (Figure 1C); Encyrtidae (11): *Acerophagus* sp1, *Acerophagus* sp2, *Aenasius arizonensis* (Girault) (Figure 1A), *Anagyrus jenniferae* Noyes & Hayat, *Anagyrus kamali* Moursi, *Anagyrus tristis* Noyes & Hayat, *Cheiloneurus nankingensis* Li & Xu (Figure 1B), *Gyranusoidea indica* Shafee, Alam & Agarwal, *Prochiloneurus javanicus* (Ferriere), *Prochiloneurus stenopterus* Wang, Huang & Xu, *Prochiloneurus testaceus* (Agarwal); Platygasteridae (1): *Allotropa phenacocca* Chen, Liu & Xu; Signiphoridae (3): *Acerophagus* sp1, *Acerophagus* sp2, *Acerophagus* sp3. (Table S1, Figure 2). Apparently, Encyrtidae species are predominant, comprising 61% of the parasitoid community found in southern China. The present study generated DNA sequences for 212 specimens, with matrices that ranged from 468 to 582 bp for 28S, and from 561 to 618 bp for COI.

### 3.1. BLAST in the NCBI and the Barcode of Life Database (BOLD)

The results of the sequence comparison against the known sequences in the BOLD system (only COI) and GenBank database (both 28S and COI) are listed in Table S4. The COI sequences of four species received “top hits” with different extents of similarity (over 98%) through the BOLD identification system. When blasted in GenBank database, four species returned close matches for COI, seven species returned close matches for 28S. Other species had no matching sequences in the BOLD or the NCBI.

### 3.2. Genetic Distances

The K2P distances (Tables S5–S8) indicated a larger intergroup than intragroup distance for both COI and 28S, and the mean genetic distance accrete with the improvement of classification. The mean intraspecific pairwise distance for COI was 0.33% (range 0–2.33%) and 0.02% (range 0–0.17%) for 28S; the mean interspecific pairwise distance for COI was 20.30% (range 9.38–35.53%) and 20.42% for 28S (range 3.25–39.04%). The mean intrageneric pairwise distance was 2.75% (range 0–8.73%) for COI and 1.50% (range 0–7.01%) for 28S; the mean intergeneric pairwise distance for COI was 21.62% (range 11.89–35.53%) and 22.03% (range 12.09–39.04%) for 28S. The mean intrafamily pairwise distance for COI was 9.38% (range 1.07–14.90%) and 7.60% (range 0.02–13.45%) for 28S; the mean interfamily pairwise distance for COI was 25.52% (range 18.11–33.67%) and 25.98% (range 16.13–36.75%) for 28S.

### 3.3. The Automatic Barcode Gap Discovery (ABGD) Analysis and Bayesian Poisson Tree Processes Model (bPTP)

The ABGD analyses returned a total of 18 groups both for 28S and COI at a priori genetic distance thresholds of 0.04–0.09, which are congruent with our identification based on morphology (Figure 2). After removing the identical sequences, 22 ingroup taxa of the concatenated data set (28S + COI) were included to construct the ML tree. The bPTP analysis based on this ML tree delimited 18 putative species, which are also congruent with the morphological identification results (Figure 2).

The 18 species of parasitoids we identified and described below.

#### 3.3.1. Aphelinidae

##### *Genus* *Marietta*

Morphological characters and molecular data both strongly support the identity of *Marietta picta* (André) (emerged from *P. solenopsis* feeding on *Justicia gendarussa* Burm. f. (Acanthaceae), *Hibiscus rosa-sinensis* L. (Malvaceae) and *Solanum nigrum* L. (Solanaceae) in Guangxi and Fujian) in this study. This species has been recorded as parasitoids of *A. arizonensis* and *Prochiloneurus aegyptiacus* (Mercet, 1929) and hyperparasitoid of *P. solenopsis* [28].

##### *Genus* *Myiocnema*

*Myiocnema comperei* Ashmead (emerged from *P. solenopsis* feeding on *Ageratum conyzoides* L. (Asteraceae), *J. gendarussa*, *H. rosa-sinensis* and *S. nigrum* in Guangxi, Fujian and Hainan) was recognized based on morphological examination and molecular analyses. The COI sequence of this species showed a more than 98% identity to *Myiocnema comperei* Ashmead in the BOLD and the NCBI. This species has been recorded as hyperparasitoids of the primary parasitoids (unidentified) of Coccidae and Pseudococcidae from China [48].

##### *Genus* *Promuscidea*

Morphological characters and molecular data, in this study, both strongly support the identity of *Promuscidea unfasciativentris* Girault (emerged from *P. solenopsis* feeding on *H. rosa-sinensis* and *S. nigrum* in Hainan). This species has been recorded as a hyperparasitoid of *P. solenopsis* [49].

#### 3.3.2. Encyrtidae

##### *Genus* *Acerophagus*

Two species of *Acerophagus* are recognized, which we temporarily named as *Acerophagus* sp1 (emerged from *P. solenopsis* feeding on *J. gendarussa* in Guangdong) and *Acerophagus* sp2 (emerged from *P. solenopsis* feeding on *H. rosa-sinensis* and *Chaenomeles sinensis* (Thouin) Koehne (Rosaceae) in Hainan and Yunnan, respectively); only the 28S sequence of *Acerophagus* sp1 showed 99.8% identity to an undetermined *Acerophagus* species in the NCBI. *Acerophagus coccois* Smith was previously reared as the primary parasitoid of *P. solenopsis* in southern China [20,27]. However, none of the two species recognized in the present study is conspecific with *Ac. coccois*. Further taxonomic work is required for both species.

##### *Genus* *Aenasius*

In this study, only one species of *Aenasius*, *A. arizonensis*, is easy to recognize by its morphology, and the COI sequences of representative specimens matched with those of *Aenasius arizonensis* (Girault) (=*Aenasius bambawalei* Hayat) both in the BOLD and in the NCBI (Table S4). *Aenasius arizonensis* seems to be the dominant primary parasitoid of *P. solenopsis* in China [27,50,51]. In our study, specimens of *A. arizonensis* comprise 56% of the total number of all parasitoid species and this species emerged from *P. solenopsis* feeding on 11 plant species in all the nine provinces surveyed. Abundant studies have shown that *A. arizonensis* is a potent parasitoid against *P. solenopsis* [10]. Developing efficient mass rearing and releasing methods could be the focus to use this species as a biological agent in future research.

##### *Genus* *Anagyrus*

The following three species of *Anagyrus* are recognized: *An. jenniferae* (emerged from *H. rosa-sinensis* in Guangdong, Guangxi, and Hainan), *An. kamali* (emerged from *P. solenopsis* feeding on *H. rosa-sinensis* in Hainan) and *An. tristis* (emerged from *P. solenopsis* feeding on *H. rosa-sinensis* and *Ficus microcarpa* L. (Moraceae) in Hainan). The ABGD and bPTP analyses also both supported a three-species scenario among the studied specimens. For the blast in the BOLD and in the NCBI, only the 28S of *An. kamali* recovered a high match in the NCBI. A number of *Anagyrus* species have been reported to attack *P. solenopsis* [19,27,28,52]. Considering the extreme morphological similarity among these species, a DNA barcode reference library of these species should enhance their accurate identifications in future surveys.

##### *Genus* *Cheiloneurus*

The morphological characters and molecular data both indicate that one species of Cheiloneurus (emerged from *P. solenopsis* feeding on *Achyranthes bidentata* Blume (Amaranthaceae), *Ag. conyzoides*, *H. rosa-sinensis* and *Portulaca grandiflora* Hook (Portulacaceae) in Fujian and Jiangxi) is present in this study. We identified the species as *Cheiloneurus nankingensis* Li & Xu based on morphology. This species was first found from Guangxi (then reported as an undescribed species, confirmed by HYC) and was erroneously reposted as a primary and dominant parasitoid of *P. solenopsis* in China. Recently, Li et al. [29] described *C. nankingensis* as a new species and confirmed that it is a hyperparasitoid with *A. arizonensis* as the host. Preliminary observation shows that this species is a gregarious parasitoid, a few and up to 18 individuals have been found emerged from a single mummy of *P. solenopsis* [27,29]. The astonishing reproduction capacity and high parasitism rate of *A. arizonensis*, renders that *C. nankingensis* might be an important destructive factor to the application of *A. arizonensis* in biological programs. In addition to this study, *C. nankingensis* has been reported from southern to eastern China. The prevalence of hyperparasitism by *C. nankingensis* across the range of *P. solenopsis* requires further investigation [29].

##### *Genus Gyranusoidea* 

The morphological and molecular data both indicate that only one species of *Gyranusoidea* (emerged from *P. solenopsis* feeding on *H. rosa-sinensis* in Hainan) is present. We identified the species as *Gyranusoidea indica* Shafee, Alam & Agarwal based on morphology. There was no blast match recovered in the BOLD or in the NCBI. *Gyranusoidea indica* has been recorded as a parasitoid of several other mealybug species and used in biological control programs against the pink hibiscus mealybug [53]. In this study, for the first time, we record this species as a parasitoid of *P. solenopsis*.

##### *Genus* *Prochiloneurus*

The following three species of *Prochiloneurus* are recognized: *Pr. javanicus* (emerged from *P. solenopsis* feeding on *H. rosa-sinensis* in Guangxi, Yunnan and Hainan), *Pr. testaceus* (emerged from *P. solenopsis* feeding on *H. rosa-sinensis* in Yunnan) and *Pr. stenopterus* (emerged from *P. solenopsis* feeding on *Ag. conyzoides* in Fujian). In addition, the ABGD and bPTP analyses both support a three-species scenario among the studied specimens, but there was no blast match recovered in the BOLD or in the NCBI. Species of *Prochiloneurus* are mainly hyperparasitoids of Coccidae and Pseudococcidae (Hemiptera) via other Encyrtidae species [54,55,56,57,58]. *Prochiloneurus stenopterus* has been previously recorded as a parasitoid of *A. arizonensis* in China [58]. *Prochiloneurus* *javanicus* and *Pr. testaceus* have been reported as hyperparasitoids of *An. kamali* [59] and *Anagyrus dactylopii* (Howard) [60], respectively.

#### 3.3.3. Platygasteridae

##### *Genus* *Allotropa*

The morphological characters and molecular data both indicate that only one species of *Allotropa* (emerged from *P. solenopsis* feeding on *H. rosa-sinensis* in Guangxi and Hainan) is present and we identify the species as *Allotropa phenacocca* Chen, Liu & Xu based on morphology. There was no blast match recovered in the BOLD or in the NCBI. This species has been reported as an endoparasitoid of *P. solenopsis* from China [21,27].

#### 3.3.4. Signiphoridae

##### *Genus* *Chartocerus*

Three species of *Chartocerus* are recognized, which we temporarily named *Chartocerus* sp1 (emerged from *P. solenopsis* feeding on *H. rosa-sinensis* in Guangxi), *Chartocerus* sp2 (emerged from *P. solenopsis* feeding on *Ag. conyzoides* in Fujian), and *Chartocerus* sp3 (emerged from *P. solenopsis* feeding on *Ag. conyzoides* in Fujian). The 28S sequences of *Chartocerus* sp1 and *Chartocerus* sp2 showed more than 98% identity to undetermined *Chartocerus* species in the NCBI. The 28S sequences of *Chartocerus* sp1 and the COI sequences of all three species showed 90–92% identity to undetermined *Chartocerus* species in the NCBI. *Chartocerus* is a relatively large genus, with more than 30 described species worldwide and four species have been recorded from China. Further taxonomic work is required for these three species. Species of *Chartocerus* are mainly known as hyperparasitoids of scale insects, mealybugs, and whiteflies (Hemiptera, Sternorrhyncha) through encyrtid or aphelinid primary parasitoids [61]. Our current study cannot verify their primary hosts in China, further studies on their biology should be carried out in the future.

**Table 1:** Hymenoptera parasitoids associated with *P. solenopsis* (the symbol “?” indicates that the biology is uncertain).

Family	Species	Parasitoid or Hyperparasitoid	Reference
Aphelinidae	*Marietta picta* (André, 1878)	parasitoid	[27,28]
	*Myiocnema comperei* Ashmead	parasitoid	[27]
	*Promuscidea unfasciativentris* Girault	hyperparasitoid	[19,27]
Encyrtidae	*Aenasius arizonensis* (Girault)	parasitoid	[18]
	*Acerophagus coccois* Smith	parasitoid	[20,27]
	*Acerophagus* sp1	parasitoid	This study
	*Acerophagus* sp2	parasitoid	This study
	*Anagyrus kamali* Moursi	parasitoid	[19,27]
	*Anagyrus agraensis* Sarawat	parasitoid	[28]
	*Anagyrus aligarhensis* Agarwal and Alam	parasitoid	[28]
	*Anagyrus californicus* (Compere)	parasitoid	[52]
	*Anagyrus dactylopii* (Howard)	parasitoid	[19]
	*Anagyrus jenniferae* Noyes & Hayat	parasitoid	This study
	*Anagyrus kamali* Moursi	parasitoid	This study
	*Anagyrus mirzai* Agarwal & Alam	parasitoid	[19]
	*Anagyrus osmoi* Guerrieri & Ghahri	parasitoid	[62]
	*Anagyrus tristis* Noyes & Hayat	parasitoid	This study
	*Bothriothorax serratellus* (Dalman)	hyperparasitoid	[28]
	*Cheiloneurus* sp.	parasitoid	[17]
	*Cheiloneurus nankingensis* Li & Xu	hyperparasitoid	[27,29]
	*Encyrtus aurantii* (Geoffroy)	parasitoid	[19]
	*Gyranusoidea indica* Shafee, Alam & Agarwal	parasitoid	This study
	*Homalotylus albiclavatus* (Agarwal)	parasitoid	[19]
	*Leptomastix algirica* Trjapitzin	parasitoid	[52]
	*Leptomastix dactylopii* Howard	parasitoid	[28]
	*Leptomastix mayri* Ózdikmen	parasitoid	[28]
	*Leptomastix epona* (Walker)	parasitoid	[26]
	*Metaphycus* sp.	parasitoid	[22]
	*Prochiloneurus aegyptiacus* (Mercet)	hyperparasitoid	[28]
	*Prochiloneurus javanicus* (Ferriere)	hyperparasitoid	[63]; This study
	*Prochiloneurus nagasakiensis* (Ishii)	hyperparasitoid	[20]
	*Prochiloneurus nigricornis* (Girault)	hyperparasitoid	[27]
	*Prochiloneurus pulchellus* Silvestri	hyperparasitoid	[19,58]
	*Prochiloneurus rex* (Girault)	hyperparasitoid	[28]
	*Prochiloneurus stenopterus* Wang, Huang & Xu	hyperparasitoid	[58]; This study
	*Prochiloneurus testaceus* (Agarwal)	hyperparasitoid	This study
	*Prochiloneurus uyguni* Hayat	hyperparasitoid	[26]
	*Prochiloneurus* sp.	? hyperparasitoid	This study
	*Pseudleptomastix squammulata* Girault	parasitoid	[52]
Eulophidae	*Aprostocetus bangaloricus* Narendran	parasitoid	[19]
	*Aprostocetus minutus* (Howard)	parasitoid	[17]
Platygastridae	*Allotropa phenacocca* Chen, Liu & Xu	parasitoid	[21,27]
Pteromalidae	*Pachyneuron leucopiscida* Mani	parasitoid	[19]
Signiphoridae	*Chartocerus kerrichi* (Agarwal)	parasitoid	[19]
	*Chartocerus kurdjumovi* (Nikolskaya)	hyperparasitoid	[28]
	*Chartocerus* sp1	?hyperparasitoid	This study
	*Chartocerus* sp2	?hyperparasitoid	This study
	*Chartocerus* sp3	?hyperparasitoid	This study

## 4. Conclusions

The parasitoid wasps associated with *P. solenopsis* from China are diverse. Our analyses based on both morphology and molecular data revealed 18 species belonging to 11 genera of four Hymenoptera families. Eight species of these wasps are primary parasitoids of *P. solenopsis* and some dominant species such as *A. arizonensis* have the potential to be developed as potent biological agents, while the other 10 species are hyperparasitoids such as *C. nankingensis*, which may diminish the parasitism of the primary parasitoids of *P. solenopsis*. The integrated taxonomic approach applied in the present study to investigate the parasitoid community associated with *P. solenopsis* should enhance the accurate identification of these parasitoids, which is of great importance for developing an effective biological control program for this invasive pest in China. Moreover, this study contributes to further understanding some of the ecological interactions between the invasive *P. solenopsis* and the native associated parasitoids.

## Data Availability

The data of the research were deposited in the Institute of Zoology, Chinese Academy of Scineces (IZCAS).

## Supplementary Materials

The following are available online at https://www.mdpi.com/article/10.3390/insects12040290/s1. Table S1. Details of the sampling, host plants and number of parasitoids collected; Table S2. Details of the sampling and accession numbers of parasitoids sequenced; Table S3. Primers used to amplify two loci in this study; Table S4. Results of molecular analyses by ABGD, bPTP, BOLD (Barcode of Life Database) blast and NCBI blast (target species with similarity value less than 97% are not shown); Table S5. Genetic distances between parasitoid species between 28S sequences under K2P model; Table S6. Genetic distances between parasitoid species between COI sequences under K2P model; Table S7. Genetic distance within parasitoid species under K2P model; Table S8 K2P pairwise distances (%) of the COI and 28S gene within different taxonomic levels of the investigated parasitoids.
