# Supplementary material for: Investigating the Parasitoid Community Associated with the Invasive Mealybug Phenacoccus solenopsis in Southern China
Source: Insects. 2021 Mar 26;12(4):290. doi: 10.3390/insects12040290 (PMC8066467; doi:10.3390/insects12040290)
Supplement: Supplementary file 1 [file insects-12-00290-s001.pdf]

Supplementary material

Investigating the parasitoid community associated with the invasive mealybug *Phenacoccus solenopsis* in southern China

Hua-Yan Chen[1,2], Hong-Liang Li[1], Hong Pang[2], Chao-Dong Zhu[1], Yan-Zhou Zhang[1*]

1 Key Laboratory of Zoological Systematics and Evolution, Institute of Zoology, Chinese Academy of Sciences, Beijing (e-mail: 454929670@qq.com; zhucd@ioz.ac.cn)
2 *State Key Laboratory of Biocontrol, Ecology and Evolution, School of Life Sciences, Sun Yat-sen University, Guangzhou, 510275, PR China* (e-mail: chenhuayan@mail.sysu.edu.cn; Lsshpang@mail.sysu.edu.cn)
[*]Corresponding author: Yan-Zhou Zhang (zhangyz@ioz.ac.cn)

Table S1. Details of the sampling, host plants and number of parasitoids collected

Species	Locality (Province, City)	Callection date	Host plant	No. parasitoids
Acerophagus sp1	Guangdong, Zhongshan	7.v.2015	*Justicia gendarussa* Burm. f.	3
Acerophagus sp2	Hainan, Sanya	21.ix.2015	*Hibiscus rosa-sinensis* L.	32
Acerophagus sp2	Hainan, Sanya	21.ix.2015	*Chaenomeles sinensi*s (Thouin) Koehne	23
Acerophagus sp2	Yunnan, Mengla	13.x.2016	*Hibiscus rosa-sinensis* L.	10
Aenasius arizonensis (Girault)	Guangdong, Zhongshan	viii.2010	*Hibiscus rosa-sinensis* L.	3
Aenasius arizonensis (Girault)	Jiangxi, Nanchang	12.x.2012	*Hibiscus rosa-sinensis* L.	4
Aenasius arizonensis (Girault)	Hainan, Wenchang	8.vii.2014	*Hibiscus rosa-sinensis* L.	4
Aenasius arizonensis (Girault)	Guangxi, Beihai	2.v.2015	*Wedelia chinensis* (Osbeck.) Merr.	20
Aenasius arizonensis (Girault)	Guangxi, Yizhou	21.vii.2015	*Justicia gendarussa* Burm. f.	33
Aenasius arizonensis (Girault)	Guangdong, Zhongshan	7.v.2015	*Justicia gendarussa* Burm. f.	23
Aenasius arizonensis (Girault)	Guangdong, Zhongshan	8.v.2015	*Hibiscus rosa-sinensis* L.	12
Aenasius arizonensis (Girault)	Hainan, Sanya	21.ix.2015	*Hibiscus rosa-sinensis* L.	3
Aenasius arizonensis (Girault)	Hainan, Haikou	27.viii.2016	*Hibiscus rosa-sinensis* L.	8
Aenasius arizonensis (Girault)	Yunnan, Mengla	13.x.2016	*Hibiscus rosa-sinensis* L.	110
Aenasius arizonensis (Girault)	Zhejiang, Hangzhou	2.viii.2016	*Justicia gendarussa* Burm. f.	5
Aenasius arizonensis (Girault)	Zhejiang, Wuyi	7.viii.2017	*Sida* sp.	5
Aenasius arizonensis (Girault)	Fujian, Xianyou	11.viii.2017	*Solanum nigrum* L.	6
Aenasius arizonensis (Girault)	Fujian, Xianyou	11.viii.2017	*Ageratum conyzoides* L.	200
Aenasius arizonensis (Girault)	Fujian, Xiamen	14.viii.2017	*Ageratum conyzoides* L.	5
Aenasius arizonensis (Girault)	Fujian, Xiamen	12.viii.2017	*Hibiscus rosa-sinensis* L.	5

Aenasius arizonensis (Girault)	Jiangxi, Ganzhou	17.viii.2017	*Portulaca grandiflora* Hook	5
Aenasius arizonensis (Girault)	Hunan, Changsha	18.viii.2017	*Achyranthes bidentata* Blume	11
Aenasius arizonensis (Girault)	Hunan, Yueyang	19.viii.2017	*Acalypha australis* L.	11
Aenasius arizonensis (Girault)	Hubei, Wuhan	22.viii.2017	*Achyranthes bidentata* Blume	11
Aenasius arizonensis (Girault)	Zhejiang, Wenzhou	30.v.2015	*Solanum nigrum* L.	5
Anagyrus jenniferae Noyes & Hayat	Hainan, Wenchang	8.vii.2014	*Hibiscus rosa-sinensis* L.	2
Anagyrus jenniferae Noyes & Hayat	Guangxi, Beihai	2.v.2015	*Hibiscus rosa-sinensis* L.	5
Anagyrus jenniferae Noyes & Hayat	Guangdong, Zhongshan	7.v.2015	*Hibiscus rosa-sinensis* L.	13
Anagyrus jenniferae Noyes & Hayat	Hainan, Haikou	22.viii.2016	*Hibiscus rosa-sinensis* L.	4
Anagyrus kamali Moursi	Hainan, Haikou	22.viii.2016	*Hibiscus rosa-sinensis* L.	5
Anagyrus tristis Noyes & Hayat	Hainan, Sanya	30.i.2013	*Ficus microcarpa* Linn. f.	28
Anagyrus tristis Noyes & Hayat	Hainan, Wenchang	8.vii.2014	*Hibiscus rosa-sinensis* L.	7
Cheiloneurus nankingensis Li & Xu	Fujian, Xianyou	11.viii.2017	*Achyranthes bidentata* Blume	6
Cheiloneurus nankingensis Li & Xu	Fujian, Xiamen	14.viii.2017	*Ageratum conyzoides* L.	2
Cheiloneurus nankingensis Li & Xu	Fujian, Xiamen	12.viii.2017	*Hibiscus rosa-sinensis* L.	6
Cheiloneurus nankingensis Li & Xu	Jiangxi, Ganzhou	17.viii.2017	*Portulaca grandiflora* Hook	5
Gyranusoidea indica Shafee, Alam & Agarwal	Hainan, Haikou	22.viii.2016	*Hibiscus rosa-sinensis* L.	2
Prochiloneurus javanicus (Ferriere)	Guangxi, Beihai	2.v.2015	*Hibiscus rosa-sinensis* L.	4
Prochiloneurus javanicus (Ferriere)	Hainan, Sanya	21.ix.2015	*Hibiscus rosa-sinensis* L.	5
Prochiloneurus javanicus (Ferriere)	Hainan, Haikou	22.viii.2016	*Hibiscus rosa-sinensis* L.	30
Prochiloneurus javanicus (Ferriere)	Yunnan, Mengla	13.x.2016	*Hibiscus rosa-sinensis* L.	4
Prochiloneurus testaceus (Agarwal)	Yunnan, Mengla	13.x.2016	*Hibiscus rosa-sinensis* L.	3
Prochiloneurus stenopterus Wang, Huang & Xu	Fujian, Xianyou	11.viii.2017	*Ageratum conyzoides* L.	3
Prochiloneurus stenopterus Wang, Huang & Xu	Fujian, Xiamen	14.viii.2017	*Ageratum conyzoides* L.	2
Marietta picta (André)	Guangxi, Yizhou	17.vii.2015	*Justicia gendarussa* Burm. f.	4

Marietta picta (André)	Fujian, Xianyou	11.viii.2017	*Solanum nigrum* L.	5
Marietta picta (André)	Fujian, Xianyou	11.viii.2017	*Achyranthes bidentata* Blume	3
Marietta picta (André)	Fujian, Xiamen	12.viii.2017	*Hibiscus rosa-sinensis* L.	2
Marietta picta (André)	Hubei, Wuhan	22.viii.2017	*Achyranthes bidentata* Blume	5
Myiocnema comperei Ashmead	Guangxi, Yizhou	17.vii.2015	*Justicia gendarussa* Burm. f.	5
Myiocnema comperei Ashmead	Hainan, Haikou	22.viii.2016	*Hibiscus rosa-sinensis* L.	10
Myiocnema comperei Ashmead	Fujian, Xianyou	11.viii.2017	*Solanum nigrum* L.	5
Myiocnema comperei Ashmead	Fujian, Xianyou	11.viii.2017	*Achyranthes bidentata* Blume	3
Myiocnema comperei Ashmead	Fujian, Xiamen	14.viii.2017	*Ageratum conyzoides* L.	3
Myiocnema comperei Ashmead	Fujian, Xiamen	12.viii.2017	*Hibiscus rosa-sinensis* L.	5
Promuscidea unfasciativentris Girault	Hainan, Haikou	22.viii.2016	*Hibiscus rosa-sinensis* L.	80
Chartocerus sp1	Guangxi, Beihai	2.v.2015	*Hibiscus rosa-sinensis* L.	3
Chartocerus sp2	Fujian, Xianyou	11.viii.2017	*Ageratum conyzoides* L.	5
Chartocerus sp3	Fujian, Xianyou	11.viii.2017	*Ageratum conyzoides* L.	7
Allotropa phenacocca Chen, Liu & Xu	Guangxi, Beihai	2.v.2015	*Hibiscus rosa-sinensis* L.	4
Allotropa phenacocca Chen, Liu & Xu	Guangdong, Zhongshan	7.v.2015	*Hibiscus rosa-sinensis* L.	3
Allotropa phenacocca Chen, Liu & Xu	Hainan, Haikou	22.viii.2016	*Hibiscus rosa-sinensis* L.	25

Table S2. Details of the sampling and accession numbers of parasitoids sequenced

					GenBank accession number	
Code	Species	Locality (Province, City)	Callection date	Host plant	28S	COI
E5-212A	*Acerophagus* sp1	Guangdong, Zhongshan	7.v.2015	*Justicia gendarussa* Burm. f.	MT783468	MT775601
E5-212B	*Acerophagus* sp1	Guangdong, Zhongshan	7.v.2015	*Justicia gendarussa* Burm. f.	MT783469	MT775602
E5-212C	*Acerophagus* sp1	Guangdong, Zhongshan	7.v.2015	*Justicia gendarussa* Burm. f.	MT783470	MT775603
E5-321A	*Acerophagus* sp2	Hainan, Sanya	21.ix.2015	*Hibiscus rosa-sinensis* L.	MT783471	MT775604
E5-321B	*Acerophagus* sp2	Hainan, Sanya	21.ix.2015	*Hibiscus rosa-sinensis* L.	MT783472	MT775605
E5-321C	*Acerophagus* sp2	Hainan, Sanya	21.ix.2015	*Hibiscus rosa-sinensis* L.	MT783473	MT775606
E5-325A	*Acerophagus* sp2	Hainan, Sanya	21.ix.2015	*Chaenomeles sinensi*s (Thouin) Koehne	MT783474	MT775607
E5-325B	*Acerophagus* sp2	Hainan, Sanya	21.ix.2015	*Chaenomeles sinensi*s (Thouin) Koehne	MT783475	MT775608
E5-325C	*Acerophagus* sp2	Hainan, Sanya	21.ix.2015	*Chaenomeles sinensi*s (Thouin) Koehne	MT783476	MT775609
E6-175A	*Acerophagus* sp2	Yunnan, Mengla	13.x.2016	*Hibiscus rosa-sinensis* L.	MT783477	MT775610
E6-175B	*Acerophagus* sp2	Yunnan, Mengla	13.x.2016	*Hibiscus rosa-sinensis* L.	MT783478	MT775611
E6-175C	*Acerophagus* sp2	Yunnan, Mengla	13.x.2016	*Hibiscus rosa-sinensis* L.	MT783479	MT775612
E6-175D	*Acerophagus* sp2	Yunnan, Mengla	13.x.2016	*Hibiscus rosa-sinensis* L.	MT783480	MT775613
10-016	*Aenasius arizonensis* (Girault)	Guangdong, Zhongshan	viii.2010	*Hibiscus rosa-sinensis* L.	MT783481	MT775614
10-017	*Aenasius arizonensis* (Girault)	Guangdong, Zhongshan	viii.2010	*Hibiscus rosa-sinensis* L.	MT783482	MT775615
E2-104A	*Aenasius arizonensis* (Girault)	Jiangxi, Nanchang	12.x.2012	*Hibiscus rosa-sinensis* L.	MT783483	MT775616
E2-104B	*Aenasius arizonensis* (Girault)	Jiangxi, Nanchang	12.x.2012	*Hibiscus rosa-sinensis* L.	MT783484	MT775617
E4-063A	*Aenasius arizonensis* (Girault)	Hainan, Wenchang	8.vii.2014	*Hibiscus rosa-sinensis* L.	MT783485	MT775618
E4-063B	*Aenasius arizonensis* (Girault)	Hainan, Wenchang	8.vii.2014	*Hibiscus rosa-sinensis* L.	MT783486	MT775619
E5-105A	*Aenasius arizonensis* (Girault)	Guangxi, Beihai	2.v.2015	*Wedelia chinensis* (Osbeck.) Merr.	MT783487	MT775620

E5-105B	*Aenasius arizonensis* (Girault)	Guangxi, Beihai	2.v.2015	*Wedelia chinensis* (Osbeck.) Merr.	MT783488	MT775621
E5-105C	*Aenasius arizonensis* (Girault)	Guangxi, Beihai	2.v.2015	*Wedelia chinensis* (Osbeck.) Merr.	MT783489	MT775622
E5-114A	*Aenasius arizonensis* (Girault)	Guangxi, Yizhou	21.vii.2015	*Justicia gendarussa* Burm. f.	MT783490	MT775623
E5-114B	*Aenasius arizonensis* (Girault)	Guangxi, Yizhou	21.vii.2015	*Justicia gendarussa* Burm. f.	MT783491	MT775624
E5-114C	*Aenasius arizonensis* (Girault)	Guangxi, Yizhou	21.vii.2015	*Justicia gendarussa* Burm. f.	MT783492	MT775625
E5-114D	*Aenasius arizonensis* (Girault)	Guangxi, Yizhou	21.vii.2015	*Justicia gendarussa* Burm. f.	MT783493	MT775626
E5-114E	*Aenasius arizonensis* (Girault)	Guangxi, Yizhou	21.vii.2015	*Justicia gendarussa* Burm. f.	MT783494	MT775627
E5-211A	*Aenasius arizonensis* (Girault)	Guangdong, Zhongshan	7.v.2015	*Justicia gendarussa* Burm. f.	MT783495	MT775628
E5-211B	*Aenasius arizonensis* (Girault)	Guangdong, Zhongshan	7.v.2015	*Justicia gendarussa* Burm. f.	MT783496	MT775629
E5-211C	*Aenasius arizonensis* (Girault)	Guangdong, Zhongshan	7.v.2015	*Justicia gendarussa* Burm. f.	MT783497	MT775630
E5-214A	*Aenasius arizonensis* (Girault)	Guangdong, Zhongshan	8.v.2015	*Hibiscus rosa-sinensis* L.	MT783498	MT775631
E5-214B	*Aenasius arizonensis* (Girault)	Guangdong, Zhongshan	8.v.2015	*Hibiscus rosa-sinensis* L.	MT783499	MT775632
E5-214C	*Aenasius arizonensis* (Girault)	Guangdong, Zhongshan	8.v.2015	*Hibiscus rosa-sinensis* L.	MT783500	MT775633
E5-320A	*Aenasius arizonensis* (Girault)	Hainan, Sanya	21.ix.2015	*Hibiscus rosa-sinensis* L.	MT783501	MT775634
E5-320B	*Aenasius arizonensis* (Girault)	Hainan, Sanya	21.ix.2015	*Hibiscus rosa-sinensis* L.	MT783502	MT775635
E6-058A	*Aenasius arizonensis* (Girault)	Hainan, Haikou	27.viii.2016	*Hibiscus rosa-sinensis* L.	MT783503	MT775636
E6-174A	*Aenasius arizonensis* (Girault)	Yunnan, Mengla	13.x.2016	*Hibiscus rosa-sinensis* L.	MT783504	MT775637
E6-174B	*Aenasius arizonensis* (Girault)	Yunnan, Mengla	13.x.2016	*Hibiscus rosa-sinensis* L.	MT783505	MT775638
E6-174C	*Aenasius arizonensis* (Girault)	Yunnan, Mengla	13.x.2016	*Hibiscus rosa-sinensis* L.	MT783506	MT775639
E6-174D	*Aenasius arizonensis* (Girault)	Yunnan, Mengla	13.x.2016	*Hibiscus rosa-sinensis* L.	MT783507	MT775640
E7-221A	*Aenasius arizonensis* (Girault)	Zhejiang, Hangzhou	2.viii.2016	*Justicia gendarussa* Burm. f.	MT783508	MT775641
E7-221B	*Aenasius arizonensis* (Girault)	Zhejiang, Hangzhou	2.viii.2016	*Justicia gendarussa* Burm. f.	MT783509	MT775642
E7-221C	*Aenasius arizonensis* (Girault)	Zhejiang, Hangzhou	2.viii.2016	*Justicia gendarussa* Burm. f.	MT783510	MT775643
E7-221D	*Aenasius arizonensis* (Girault)	Zhejiang, Hangzhou	2.viii.2016	*Justicia gendarussa* Burm. f.	MT783511	MT775644
E7-223A	*Aenasius arizonensis* (Girault)	Zhejiang, Wuyi	7.viii.2017	*Sida* sp.	MT783512	MT775645
E7-223B	*Aenasius arizonensis* (Girault)	Zhejiang, Wuyi	7.viii.2017	*Sida* sp.	MT783513	MT775646

E7-223C	*Aenasius arizonensis* (Girault)	Zhejiang, Wuyi	7.viii.2017	*Sida* sp.	MT783514	MT775647
E7-223D	*Aenasius arizonensis* (Girault)	Zhejiang, Wuyi	7.viii.2017	*Sida* sp.	MT783515	MT775648
E7-244A	*Aenasius arizonensis* (Girault)	Fujian, Xianyou	11.viii.2017	*Solanum nigrum* L.	MT783516	MT775649
E7-244B	*Aenasius arizonensis* (Girault)	Fujian, Xianyou	11.viii.2017	*Solanum nigrum* L.	MT783517	MT775650
E7-244C	*Aenasius arizonensis* (Girault)	Fujian, Xianyou	11.viii.2017	*Solanum nigrum* L.	MT783518	MT775651
E7-244D	*Aenasius arizonensis* (Girault)	Fujian, Xianyou	11.viii.2017	*Solanum nigrum* L.	MT783519	MT775652
E7-244E	*Aenasius arizonensis* (Girault)	Fujian, Xianyou	11.viii.2017	*Solanum nigrum* L.	MT783520	MT775653
E7-244F	*Aenasius arizonensis* (Girault)	Fujian, Xianyou	11.viii.2017	*Solanum nigrum* L.	MT783521	MT775654
E7-250A	*Aenasius arizonensis* (Girault)	Fujian, Xianyou	11.viii.2017	*Ageratum conyzoides* L.	MT783522	MT775655
E7-250B	*Aenasius arizonensis* (Girault)	Fujian, Xianyou	11.viii.2017	*Ageratum conyzoides* L.	MT783523	MT775656
E7-260A	*Aenasius arizonensis* (Girault)	Fujian, Xiamen	14.viii.2017	*Ageratum conyzoides* L.	MT783524	MT775657
E7-260B	*Aenasius arizonensis* (Girault)	Fujian, Xiamen	14.viii.2017	*Ageratum conyzoides* L.	MT783525	MT775658
E7-265A	*Aenasius arizonensis* (Girault)	Fujian, Xiamen	12.viii.2017	*Hibiscus rosa-sinensis* L.	MT783526	MT775659
E7-265B	*Aenasius arizonensis* (Girault)	Fujian, Xiamen	12.viii.2017	*Hibiscus rosa-sinensis* L.	MT783527	MT775660
E7-265C	*Aenasius arizonensis* (Girault)	Fujian, Xiamen	12.viii.2017	*Hibiscus rosa-sinensis* L.	MT783528	MT775661
E7-273A	*Aenasius arizonensis* (Girault)	Jiangxi, Ganzhou	17.viii.2017	*Portulaca grandiflora* Hook	MT783529	MT775662
E7-273B	*Aenasius arizonensis* (Girault)	Jiangxi, Ganzhou	17.viii.2017	*Portulaca grandiflora* Hook	MT783530	MT775663
E7-273C	*Aenasius arizonensis* (Girault)	Jiangxi, Ganzhou	17.viii.2017	*Portulaca grandiflora* Hook	MT783531	MT775664
E7-276A	*Aenasius arizonensis* (Girault)	Hunan, Changsha	18.viii.2017	*Achyranthes bidentata* Blume	MT783532	MT775665
E7-276B	*Aenasius arizonensis* (Girault)	Hunan, Changsha	18.viii.2017	*Achyranthes bidentata* Blume	MT783533	MT775666
E7-276C	*Aenasius arizonensis* (Girault)	Hunan, Changsha	18.viii.2017	*Achyranthes bidentata* Blume	MT783534	MT775667
E7-276D	*Aenasius arizonensis* (Girault)	Hunan, Changsha	18.viii.2017	*Achyranthes bidentata* Blume	MT783535	MT775668
E7-278A	*Aenasius arizonensis* (Girault)	Hunan, Yueyang	19.viii.2017	*Acalypha australis* L.	MT783536	MT775669
E7-278B	*Aenasius arizonensis* (Girault)	Hunan, Yueyang	19.viii.2017	*Acalypha australis* L.	MT783537	MT775670
E7-278C	*Aenasius arizonensis* (Girault)	Hunan, Yueyang	19.viii.2017	*Acalypha australis* L.	MT783538	MT775671
E7-278D	*Aenasius arizonensis* (Girault)	Hunan, Yueyang	19.viii.2017	*Acalypha australis* L.	MT783539	MT775672
E7-281A	*Aenasius arizonensis* (Girault)	Hubei, Wuhan	22.viii.2017	*Achyranthes bidentata* Blume	MT783540	MT775673
E7-281B	*Aenasius arizonensis* (Girault)	Hubei, Wuhan	22.viii.2017	*Achyranthes bidentata* Blume	MT783541	MT775674

E7-281C	*Aenasius arizonensis* (Girault)	Hubei, Wuhan	22.viii.2017	*Achyranthes bidentata* Blume	MT783542	MT775675
E7-281D	*Aenasius arizonensis* (Girault)	Hubei, Wuhan	22.viii.2017	*Achyranthes bidentata* Blume	MT783543	MT775676
E7-281E	*Aenasius arizonensis* (Girault)	Hubei, Wuhan	22.viii.2017	*Achyranthes bidentata* Blume	MT783544	MT775677
E4-151A	*Aenasius arizonensis* (Girault)	Hubei, Wuhan	22.viii.2017	*Achyranthes bidentata* Blume	MT783545	MT775678
E4-151B	*Aenasius arizonensis* (Girault)	Hubei, Wuhan	22.viii.2017	*Achyranthes bidentata* Blume	MT783546	MT775679
E4-151C	*Aenasius arizonensis* (Girault)	Hubei, Wuhan	22.viii.2017	*Achyranthes bidentata* Blume	MT783547	MT775680
E4-151D	*Aenasius arizonensis* (Girault)	Hubei, Wuhan	22.viii.2017	*Achyranthes bidentata* Blume	MT783548	MT775681
E5-287A	*Aenasius arizonensis* (Girault)	Zhejiang, Wenzhou	30.v.2015	*Solanum nigrum* L.	MT783549	MT775682
E5-287B	*Aenasius arizonensis* (Girault)	Zhejiang, Wenzhou	30.v.2015	*Solanum nigrum* L.	MT783550	MT775683
E5-287C	*Aenasius arizonensis* (Girault)	Zhejiang, Wenzhou	30.v.2015	*Solanum nigrum* L.	MT783551	MT775684
E4-064A	*Anagyrus jenniferae* Noyes & Hayat	Hainan, Wenchang	8.vii.2014	*Hibiscus rosa-sinensis* L.	MT783552	MT775685
E4-064B	*Anagyrus jenniferae* Noyes & Hayat	Hainan, Wenchang	8.vii.2014	*Hibiscus rosa-sinensis* L.	MT783553	MT775686
E5-103A	*Anagyrus jenniferae* Noyes & Hayat	Guangxi, Beihai	2.v.2015	*Hibiscus rosa-sinensis* L.	MT783554	MT775687
E5-103B	*Anagyrus jenniferae* Noyes & Hayat	Guangxi, Beihai	2.v.2015	*Hibiscus rosa-sinensis* L.	MT783555	MT775688
E5-103C	*Anagyrus jenniferae* Noyes & Hayat	Guangxi, Beihai	2.v.2015	*Hibiscus rosa-sinensis* L.	MT783556	MT775689
E5-215A	*Anagyrus jenniferae* Noyes & Hayat	Guangdong, Zhongshan	7.v.2015	*Hibiscus rosa-sinensis* L.	MT783557	MT775690
E5-215B	*Anagyrus jenniferae* Noyes & Hayat	Guangdong, Zhongshan	7.v.2015	*Hibiscus rosa-sinensis* L.	MT783558	MT775691
E5-215C	*Anagyrus jenniferae* Noyes & Hayat	Guangdong, Zhongshan	7.v.2015	*Hibiscus rosa-sinensis* L.	MT783559	MT775692
E6-059A	*Anagyrus jenniferae* Noyes & Hayat	Hainan, Haikou	22.viii.2016	*Hibiscus rosa-sinensis* L.	MT783560	MT775693

E6-059B	*Anagyrus jenniferae* Noyes & Hayat	Hainan, Haikou	22.viii.2016	*Hibiscus rosa-sinensis* L.	MT783561	MT775694
E6-060C	*Anagyrus jenniferae* Noyes & Hayat	Hainan, Haikou	22.viii.2016	*Hibiscus rosa-sinensis* L.	MT783562	MT775695
E6-060D	*Anagyrus jenniferae* Noyes & Hayat	Hainan, Haikou	22.viii.2016	*Hibiscus rosa-sinensis* L.	MT783563	MT775696
E6-059C	*Anagyrus kamali* Moursi	Hainan, Haikou	22.viii.2016	*Hibiscus rosa-sinensis* L.	MT783564	MT775697
E6-059D	*Anagyrus kamali* Moursi	Hainan, Haikou	22.viii.2016	*Hibiscus rosa-sinensis* L.	MT783565	MT775698
E6-059E	*Anagyrus kamali* Moursi	Hainan, Haikou	22.viii.2016	*Hibiscus rosa-sinensis* L.	MT783566	MT775699
E6-060A	*Anagyrus kamali* Moursi	Hainan, Haikou	22.viii.2016	*Hibiscus rosa-sinensis* L.	MT783567	MT775700
E6-060B	*Anagyrus kamali* Moursi	Hainan, Haikou	22.viii.2016	*Hibiscus rosa-sinensis* L.	MT783568	MT775701
E3-119A	*Anagyrus tristis* Noyes & Hayat	Hainan, Sanya	30.i.2013	*Ficus microcarpa* Linn. f.	MT783569	MT775702
E3-119B	*Anagyrus tristis* Noyes & Hayat	Hainan, Sanya	30.i.2013	*Ficus microcarpa* Linn. f.	MT783570	MT775703
E3-119C	*Anagyrus tristis* Noyes & Hayat	Hainan, Sanya	30.i.2013	*Ficus microcarpa* Linn. f.	MT783571	MT775704
E3-119D	*Anagyrus tristis* Noyes & Hayat	Hainan, Sanya	30.i.2013	*Ficus microcarpa* Linn. f.	MT783572	MT775705
E3-119E	*Anagyrus tristis* Noyes & Hayat	Hainan, Sanya	30.i.2013	*Ficus microcarpa* Linn. f.	MT783573	MT775706
E4-062A	*Anagyrus tristis* Noyes & Hayat	Hainan, Wenchang	8.vii.2014	*Hibiscus rosa-sinensis* L.	MT783574	MT775707
E4-062B	*Anagyrus tristis* Noyes & Hayat	Hainan, Wenchang	8.vii.2014	*Hibiscus rosa-sinensis* L.	MT783575	MT775708
E4-062C	*Anagyrus tristis* Noyes & Hayat	Hainan, Wenchang	8.vii.2014	*Hibiscus rosa-sinensis* L.	MT783576	MT775709
E4-062D	*Anagyrus tristis* Noyes & Hayat	Hainan, Wenchang	8.vii.2014	*Hibiscus rosa-sinensis* L.	MT783577	MT775710
E7-247A	*Cheiloneurus nankingensis* Li & Xu	Fujian, Xianyou	11.viii.2017	*Achyranthes bidentata* Blume	MT783578	MT775711
E7-247B	*Cheiloneurus nankingensis* Li & Xu	Fujian, Xianyou	11.viii.2017	*Achyranthes bidentata* Blume	MT783579	MT775712
E7-247C	*Cheiloneurus nankingensis* Li & Xu	Fujian, Xianyou	11.viii.2017	*Achyranthes bidentata* Blume	MT783580	MT775713
E7-247D	*Cheiloneurus nankingensis* Li & Xu	Fujian, Xianyou	11.viii.2017	*Achyranthes bidentata* Blume	MT783581	MT775714

E7-247E	*Cheiloneurus nankingensis* Li & Xu	Fujian, Xianyou	11.viii.2017	*Achyranthes bidentata* Blume	MT783582	MT775715
E7-261A	*Cheiloneurus nankingensis* Li & Xu	Fujian, Xiamen	14.viii.2017	*Ageratum conyzoides* L.	MT783583	MT775716
E7-261B	*Cheiloneurus nankingensis* Li & Xu	Fujian, Xiamen	14.viii.2017	*Ageratum conyzoides* L.	MT783584	MT775717
E7-266A	*Cheiloneurus nankingensis* Li & Xu	Fujian, Xiamen	12.viii.2017	*Hibiscus rosa-sinensis* L.	MT783585	MT775718
E7-266B	*Cheiloneurus nankingensis* Li & Xu	Fujian, Xiamen	12.viii.2017	*Hibiscus rosa-sinensis* L.	MT783586	MT775719
E7-266C	*Cheiloneurus nankingensis* Li & Xu	Fujian, Xiamen	12.viii.2017	*Hibiscus rosa-sinensis* L.	MT783587	MT775720
E7-274A	*Cheiloneurus nankingensis* Li & Xu	Jiangxi, Ganzhou	17.viii.2017	*Portulaca grandiflora* Hook	MT783588	MT775721
E7-274B	*Cheiloneurus nankingensis* Li & Xu	Jiangxi, Ganzhou	17.viii.2017	*Portulaca grandiflora* Hook	MT783589	MT775722
E7-274C	*Cheiloneurus nankingensis* Li & Xu	Jiangxi, Ganzhou	17.viii.2017	*Portulaca grandiflora* Hook	MT783590	MT775723
E6-057A	*Gyranusoidea indica* Shafee, Alam & Agarwal	Hainan, Haikou	22.viii.2016	*Hibiscus rosa-sinensis* L.	MT783591	MT775724
E6-057B	*Gyranusoidea indica* Shafee, Alam & Agarwal	Hainan, Haikou	22.viii.2016	*Hibiscus rosa-sinensis* L.	MT783592	MT775725
E5-104A	*Prochiloneurus javanicus* (Ferriere)	Guangxi, Beihai	2.v.2015	*Hibiscus rosa-sinensis* L.	MT783593	MT775726
E5-104B	*Prochiloneurus javanicus* (Ferriere)	Guangxi, Beihai	2.v.2015	*Hibiscus rosa-sinensis* L.	MT783594	MT775727
E5-322A	*Prochiloneurus javanicus* (Ferriere)	Hainan, Sanya	21.ix.2015	*Hibiscus rosa-sinensis* L.	MT783595	MT775728

E5-322B	*Prochiloneurus javanicus* (Ferriere)	Hainan, Sanya	21.ix.2015	*Hibiscus rosa-sinensis* L.	MT783596	MT775729
E6-061A	*Prochiloneurus javanicus* (Ferriere)	Hainan, Haikou	22.viii.2016	*Hibiscus rosa-sinensis* L.	MT783597	MT775730
E6-061B	*Prochiloneurus javanicus* (Ferriere)	Hainan, Haikou	22.viii.2016	*Hibiscus rosa-sinensis* L.	MT783598	MT775731
E6-061C	*Prochiloneurus javanicus* (Ferriere)	Hainan, Haikou	22.viii.2016	*Hibiscus rosa-sinensis* L.	MT783599	MT775732
E6-061D	*Prochiloneurus javanicus* (Ferriere)	Hainan, Haikou	22.viii.2016	*Hibiscus rosa-sinensis* L.	MT783600	MT775733
E6-061E	*Prochiloneurus javanicus* (Ferriere)	Hainan, Haikou	22.viii.2016	*Hibiscus rosa-sinensis* L.	MT783601	MT775734
E6-061F	*Prochiloneurus javanicus* (Ferriere)	Hainan, Haikou	22.viii.2016	*Hibiscus rosa-sinensis* L.	MT783602	MT775735
E6-176A	*Prochiloneurus javanicus* (Ferriere)	Yunnan, Mengla	13.x.2016	*Hibiscus rosa-sinensis* L.	MT783603	MT775736
E6-176B	*Prochiloneurus javanicus* (Ferriere)	Yunnan, Mengla	13.x.2016	*Hibiscus rosa-sinensis* L.	MT783604	MT775737
E6-177A	*Prochiloneurus testaceus* (Agarwal)	Yunnan, Mengla	13.x.2016	*Hibiscus rosa-sinensis* L.	MT783605	MT775738
E6-177B	*Prochiloneurus testaceus* (Agarwal)	Yunnan, Mengla	13.x.2016	*Hibiscus rosa-sinensis* L.	MT783606	MT775739
E6-178A	*Prochiloneurus testaceus* (Agarwal)	Yunnan, Mengla	13.x.2016	*Hibiscus rosa-sinensis* L.	MT783607	MT775740
E7-251A	*Prochiloneurus stenopterus* Wang, Huang & Xu	Fujian, Xianyou	11.viii.2017	*Ageratum conyzoides* L.	MT783608	MT775741
E7-251B	*Prochiloneurus stenopterus* Wang, Huang & Xu	Fujian, Xianyou	11.viii.2017	*Ageratum conyzoides* L.	MT783609	MT775742

E7-251C	*Prochiloneurus stenopterus* Wang, Huang & Xu	Fujian, Xianyou	11.viii.2017	*Ageratum conyzoides* L.	MT783610	MT775743
E7-261C	*Prochiloneurus stenopterus* Wang, Huang & Xu	Fujian, Xiamen	14.viii.2017	*Ageratum conyzoides* L.	MT783611	MT775744
E7-261D	*Prochiloneurus stenopterus* Wang, Huang & Xu	Fujian, Xiamen	14.viii.2017	*Ageratum conyzoides* L.	MT783612	MT775745
A5-115A	*Marietta picta* (André)	Guangxi, Yizhou	17.vii.2015	*Justicia gendarussa* Burm. f.	MT783613	MT775746
A5-115B	*Marietta picta* (André)	Guangxi, Yizhou	17.vii.2015	*Justicia gendarussa* Burm. f.	MT783614	MT775747
A5-115C	*Marietta picta* (André)	Guangxi, Yizhou	17.vii.2015	*Justicia gendarussa* Burm. f.	MT783615	MT775748
A5-115D	*Marietta picta* (André)	Guangxi, Yizhou	17.vii.2015	*Justicia gendarussa* Burm. f.	MT783616	MT775749
A7-025A	*Marietta picta* (André)	Fujian, Xianyou	11.viii.2017	*Solanum nigrum* L.	MT783617	MT775750
A7-025B	*Marietta picta* (André)	Fujian, Xianyou	11.viii.2017	*Solanum nigrum* L.	MT783618	MT775751
A7-025C	*Marietta picta* (André)	Fujian, Xianyou	11.viii.2017	*Solanum nigrum* L.	MT783619	MT775752
A7-025D	*Marietta picta* (André)	Fujian, Xianyou	11.viii.2017	*Solanum nigrum* L.	MT783620	MT775753
A7-028A	*Marietta picta* (André)	Fujian, Xianyou	11.viii.2017	*Achyranthes bidentata* Blume	MT783621	MT775754
A7-028B	*Marietta picta* (André)	Fujian, Xianyou	11.viii.2017	*Achyranthes bidentata* Blume	MT783622	MT775755
A7-028C	*Marietta picta* (André)	Fujian, Xianyou	11.viii.2017	*Achyranthes bidentata* Blume	MT783623	MT775756
A7-033A	*Marietta picta* (André)	Fujian, Xiamen	12.viii.2017	*Hibiscus rosa-sinensis* L.	MT783624	MT775757
A7-033B	*Marietta picta* (André)	Fujian, Xiamen	12.viii.2017	*Hibiscus rosa-sinensis* L.	MT783625	MT775758
A7-036A	*Marietta picta* (André)	Hubei, Wuhan	22.viii.2017	*Achyranthes bidentata* Blume	MT783626	MT775759
A7-036B	*Marietta picta* (André)	Hubei, Wuhan	22.viii.2017	*Achyranthes bidentata* Blume	MT783627	MT775760
A7-036C	*Marietta picta* (André)	Hubei, Wuhan	22.viii.2017	*Achyranthes bidentata* Blume	MT783628	MT775761
A7-036D	*Marietta picta* (André)	Hubei, Wuhan	22.viii.2017	*Achyranthes bidentata* Blume	MT783629	MT775762
A7-036E	*Marietta picta* (André)	Hubei, Wuhan	22.viii.2017	*Achyranthes bidentata* Blume	MT783630	MT775763
A5-114B	*Myiocnema comperei* Ashmead	Guangxi, Yizhou	17.vii.2015	*Justicia gendarussa* Burm. f.	MT783631	MT775764
A5-114C	*Myiocnema comperei* Ashmead	Guangxi, Yizhou	17.vii.2015	*Justicia gendarussa* Burm. f.	MT783632	MT775765
A5-114D	*Myiocnema comperei* Ashmead	Guangxi, Yizhou	17.vii.2015	*Justicia gendarussa* Burm. f.	MT783633	MT775766
A6-038A	*Myiocnema comperei* Ashmead	Hainan, Haikou	22.viii.2016	*Hibiscus rosa-sinensis* L.	MT783634	MT775767

A6-038B	*Myiocnema comperei* Ashmead	Hainan, Haikou	22.viii.2016	*Hibiscus rosa-sinensis* L.	MT783635	MT775768
A6-038C	*Myiocnema comperei* Ashmead	Hainan, Haikou	22.viii.2016	*Hibiscus rosa-sinensis* L.	MT783636	MT775769
A6-038D	*Myiocnema comperei* Ashmead	Hainan, Haikou	22.viii.2016	*Hibiscus rosa-sinensis* L.	MT783637	MT775770
A6-038E	*Myiocnema comperei* Ashmead	Hainan, Haikou	22.viii.2016	*Hibiscus rosa-sinensis* L.	MT783638	MT775771
A6-038F	*Myiocnema comperei* Ashmead	Hainan, Haikou	22.viii.2016	*Hibiscus rosa-sinensis* L.	MT783639	MT775772
A6-038G	*Myiocnema comperei* Ashmead	Hainan, Haikou	22.viii.2016	*Hibiscus rosa-sinensis* L.	MT783640	MT775773
A7-024A	*Myiocnema comperei* Ashmead	Fujian, Xianyou	11.viii.2017	*Solanum nigrum* L.	MT783641	MT775774
A7-027A	*Myiocnema comperei* Ashmead	Fujian, Xianyou	11.viii.2017	*Achyranthes bidentata* Blume	MT783642	MT775775
A7-027B	*Myiocnema comperei* Ashmead	Fujian, Xianyou	11.viii.2017	*Achyranthes bidentata* Blume	MT783643	MT775776
A7-030A	*Myiocnema comperei* Ashmead	Fujian, Xiamen	14.viii.2017	*Ageratum conyzoides* L.	MT783644	MT775777
A7-030B	*Myiocnema comperei* Ashmead	Fujian, Xiamen	14.viii.2017	*Ageratum conyzoides* L.	MT783645	MT775778
A7-030C	*Myiocnema comperei* Ashmead	Fujian, Xiamen	14.viii.2017	*Ageratum conyzoides* L.	MT783646	MT775779
A7-032A	*Myiocnema comperei* Ashmead	Fujian, Xiamen	12.viii.2017	*Hibiscus rosa-sinensis* L.	MT783647	MT775780
A7-032B	*Myiocnema comperei* Ashmead	Fujian, Xiamen	12.viii.2017	*Hibiscus rosa-sinensis* L.	MT783648	MT775781
A6-037A	*Promuscidea unfasciativentris* Girault	Hainan, Haikou	22.viii.2016	*Hibiscus rosa-sinensis* L.	MT783649	MT775782
A6-037B	*Promuscidea unfasciativentris* Girault	Hainan, Haikou	22.viii.2016	*Hibiscus rosa-sinensis* L.	MT783650	MT775783
A6-037C	*Promuscidea unfasciativentris* Girault	Hainan, Haikou	22.viii.2016	*Hibiscus rosa-sinensis* L.	MT783651	MT775784
A6-037D	*Promuscidea unfasciativentris* Girault	Hainan, Haikou	22.viii.2016	*Hibiscus rosa-sinensis* L.	MT783652	MT775785
A6-037E	*Promuscidea unfasciativentris* Girault	Hainan, Haikou	22.viii.2016	*Hibiscus rosa-sinensis* L.	MT783653	MT775786
A6-037F	*Promuscidea unfasciativentris* Girault	Hainan, Haikou	22.viii.2016	*Hibiscus rosa-sinensis* L.	MT783654	MT775787
A6-037G	*Promuscidea unfasciativentris* Girault	Hainan, Haikou	22.viii.2016	*Hibiscus rosa-sinensis* L.	MT783655	MT775788

Si5-001A	*Chartocerus* sp1	Guangxi, Beihai	2.v.2015	*Hibiscus rosa-sinensis* L.	MT783656	MT775789
Si5-001C	*Chartocerus* sp1	Guangxi, Beihai	2.v.2015	*Hibiscus rosa-sinensis* L.	MT783657	MT775790
Si7-012A	*Chartocerus* sp2	Fujian, Xianyou	11.viii.2017	*Ageratum conyzoides* L.	MT783658	MT775791
Si7-012B	*Chartocerus* sp2	Fujian, Xianyou	11.viii.2017	*Ageratum conyzoides* L.	MT783659	MT775792
Si7-012C	*Chartocerus* sp2	Fujian, Xianyou	11.viii.2017	*Ageratum conyzoides* L.	MT783660	MT775793
Si7-012D	*Chartocerus* sp2	Fujian, Xianyou	11.viii.2017	*Ageratum conyzoides* L.	MT783661	MT775794
Si7-012E	*Chartocerus* sp2	Fujian, Xianyou	11.viii.2017	*Ageratum conyzoides* L.	MT783662	MT775795
Si7-013A	*Chartocerus* sp3	Fujian, Xianyou	11.viii.2017	*Ageratum conyzoides* L.	MT783663	MT775796
Si7-013B	*Chartocerus* sp3	Fujian, Xianyou	11.viii.2017	*Ageratum conyzoides* L.	MT783664	MT775797
Si7-013C	*Chartocerus* sp3	Fujian, Xianyou	11.viii.2017	*Ageratum conyzoides* L.	MT783665	MT775798
Si7-013D	*Chartocerus* sp3	Fujian, Xianyou	11.viii.2017	*Ageratum conyzoides* L.	MT783666	MT775799
Si7-013E	*Chartocerus* sp3	Fujian, Xianyou	11.viii.2017	*Ageratum conyzoides* L.	MT783667	MT775800
Si7-013F	*Chartocerus* sp3	Fujian, Xianyou	11.viii.2017	*Ageratum conyzoides* L.	MT783668	MT775801
PL-1500A	*Allotropa phenacocca* Chen, Liu & Xu	Guangxi, Beihai	2.v.2015	*Hibiscus rosa-sinensis* L.	MT783669	MT775802
PL-1500B	*Allotropa phenacocca* Chen, Liu & Xu	Guangxi, Beihai	2.v.2015	*Hibiscus rosa-sinensis* L.	MT783670	MT775803
PL-1500C	*Allotropa phenacocca* Chen, Liu & Xu	Guangxi, Beihai	2.v.2015	*Hibiscus rosa-sinensis* L.	MT783671	MT775804
PL-1500D	*Allotropa phenacocca* Chen, Liu & Xu	Guangxi, Beihai	2.v.2015	*Hibiscus rosa-sinensis* L.	MT783672	MT775805
PL-1501A	*Allotropa phenacocca* Chen, Liu & Xu	Guangdong, Zhongshan	7.v.2015	*Hibiscus rosa-sinensis* L.	MT783673	MT775806
PL-1501B	*Allotropa phenacocca* Chen, Liu & Xu	Guangdong, Zhongshan	7.v.2015	*Hibiscus rosa-sinensis* L.	MT783674	MT775807
PL-1501C	*Allotropa phenacocca* Chen, Liu & Xu	Guangdong, Zhongshan	7.v.2015	*Hibiscus rosa-sinensis* L.	MT783675	MT775808
PL-02A	*Allotropa phenacocca* Chen, Liu	Hainan, Haikou	22.viii.2016	*Hibiscus rosa-sinensis* L.	MT783676	MT775809

	& Xu					
PL-02B	*Allotropa phenacocca* Chen, Liu & Xu	Hainan, Haikou	22.viii.2016	*Hibiscus rosa-sinensis* L.	MT783677	MT775810
PL-02C	*Allotropa phenacocca* Chen, Liu & Xu	Hainan, Haikou	22.viii.2016	*Hibiscus rosa-sinensis* L.	MT783678	MT775811
PL-02E	*Allotropa phenacocca* Chen, Liu & Xu	Hainan, Haikou	22.viii.2016	*Hibiscus rosa-sinensis* L.	MT783679	MT775812

Table S3. Primers used to amplify two loci in this study.

Gene	Primer name	Primer sequences	Reference
COI	LCO1490[F]	5'-TCTCTACTAATCATAAAATTATTGG-3'	[36]
COI	HCO-2198[R]	5'-TAAACTTCAGGGTGACCAAAAAATCA-3'	[36]
28S	D1-3317[F]	5'-ACCCGCTGAATTTAAGCATAT-3'	[37]
28S	D2-3549[F]	5'-AGTCGTGTTGCTTGATAGTGCAG-3'	[38]
28S	D2-3566[F]	5'-TGCAGCTCTAAGTTGGTGGT-3'	[39]
28S	D2-3665[F]	5'-AGAGAGAGTTCAAGAGTACGTG-3'	[40]
28S	D2-4068[R]	5'-TTGGTCCGTGTTTCAAGACGGG-3'	[38]
28S	D3-4283[R]	5'-TAGTTCACCATCTTTCGGGTC-3'	[41]

Table S4. Results of molecular analyses by ABGD, bPTP, BOLD (Barcode of Life Database) blast and NCBI blast (target species with similarity value less than 97% are not shown).

Morpholo-species	ABGD	bPTP	BOLD (COI)	NCBI	
				COI	28S
Acerophagus sp1	*Acerophagus* sp1	*Acerophagus* sp1			*Acerophagus* sp.
Acerophagus sp2	*Acerophagus* sp2	*Acerophagus* sp2			
Aenasius arizonensis	*Aenasius arizonensis*	*Aenasius arizonensis*	*Aenasius arizonensis*	*Aenasius arizonensis*	
Anagyrus jenniferae	*Anagyrus jenniferae*	*Anagyrus jenniferae*			
Anagyrus kamali	*Anagyrus kamali*	*Anagyrus kamali*			*Anagyrus kamali*
Anagyrus tristis	*Anagyrus tristis*	*Anagyrus tristis*			
Cheiloneurus nankingensis	*Cheiloneurus nankingensis*	*Cheiloneurus nankingensis*			
Gyranusoidea indica	*Gyranusoidea indica*	*Gyranusoidea indica*			
Prochiloneurus javanicus	*Prochiloneurus javanicus*	*Prochiloneurus javanicus*			
Prochiloneurus testaceus	*Prochiloneurus testaceus*	*Prochiloneurus testaceus*			
Prochiloneurus_stenopterus	*Prochiloneurus_stenopterus*	*Prochiloneurus_stenopterus*			*Cheiloneurus ceroplastis*
Marietta picta	*Marietta picta*	*Marietta picta*	*Marietta* sp.	*Marietta* sp.	*Marietta* sp.
Myiocnema comperei	*Myiocnema comperei*	*Myiocnema comperei*	*Myiocnema comperei*	*Myiocnema comperei*	
Promuscidea unfasciativentris	*Promuscidea unfasciativentris*	*Promuscidea unfasciativentris*	*Promuscidea unfasciativentris*	*Promuscidea unfasciativentris*	*Promuscidea unfasciativentris*
Chartocerus sp1	*Chartocerus* sp1	*Chartocerus* sp1		*Chartocerus* sp.	*Chartocerus* sp.
Chartocerus sp2	*Chartocerus* sp2	*Chartocerus* sp2			*Chartocerus* sp.
Chartocerus sp2	*Chartocerus* sp3	*Chartocerus* sp3			
Allotropa phenacocca	*Allotropa phenacocca*	*Allotropa phenacocca*			

Table S5. Genetic distances between parasitoid species between 28S sequences under K2P model

	Species	1	2	3	4	5	6	7	8	9	10	11	12	13	14	15	16	17	18
1	*Acerophagus* sp1		0.0085	0.0183	0.0243	0.0229	0.0229	0.0256	0.0339	0.0270	0.0271	0.0268	0.0217	0.0253	0.0223	0.0242	0.0231	0.0238	0.0322
2	*Acerophagus* sp2	0.0485		0.0184	0.0246	0.0230	0.0230	0.0269	0.0311	0.0275	0.0281	0.0273	0.0221	0.0263	0.0222	0.0244	0.0240	0.0235	0.0344
3	*Aenasius arizonensis*	0.1591	0.1587		0.0183	0.0188	0.0189	0.0209	0.0237	0.0225	0.0218	0.0220	0.0198	0.0191	0.0173	0.0197	0.0187	0.0192	0.0314
4	*Anagyrus jenniferae*	0.2247	0.2252	0.1639		0.0113	0.0139	0.0231	0.0214	0.0239	0.0237	0.0243	0.0205	0.0175	0.0208	0.0213	0.0214	0.0203	0.0324
5	*Anagyrus kamali*	0.2249	0.2247	0.1718	0.0674		0.0128	0.0230	0.0234	0.0240	0.0238	0.0232	0.0193	0.0185	0.0213	0.0215	0.0209	0.0198	0.0316
6	*Anagyrus tristis*	0.2100	0.2122	0.1498	0.1028	0.0801		0.0219	0.0204	0.0239	0.0237	0.0225	0.0188	0.0180	0.0206	0.0216	0.0197	0.0195	0.0323
7	*Cheiloneurus nankingensis*	0.2618	0.2589	0.1709	0.2037	0.2035	0.1912		0.0249	0.0165	0.0150	0.0158	0.0206	0.0194	0.0231	0.0220	0.0216	0.0228	0.0363
8	*Gyranusoidea indica*	0.3240	0.2974	0.1949	0.1745	0.1906	0.1615	0.2226		0.0262	0.0270	0.0265	0.0288	0.0244	0.0257	0.0280	0.0263	0.0243	0.0394
9	*Prochiloneurus javanicus*	0.2786	0.2844	0.2111	0.2244	0.2282	0.2220	0.1361	0.2314		0.0075	0.0086	0.0208	0.0203	0.0221	0.0242	0.0224	0.0227	0.0365
10	*Prochiloneurus testaceus*	0.2688	0.2774	0.1925	0.2145	0.2165	0.2076	0.1212	0.2373	0.0325		0.0085	0.0208	0.0199	0.0215	0.0236	0.0219	0.0228	0.0362
11	*Prochiloneurus stenopterus*	0.2604	0.2684	0.1887	0.2112	0.2107	0.1979	0.1267	0.2346	0.0441	0.0385		0.0206	0.0202	0.0209	0.0224	0.0202	0.0217	0.0363
12	*Marietta picta*	0.1907	0.1922	0.1581	0.1743	0.1623	0.1611	0.1941	0.2372	0.1934	0.1940	0.1934		0.0182	0.0212	0.0209	0.0212	0.0208	0.0333
13	*Myiocnema comperei*	0.2057	0.2129	0.1370	0.1255	0.1379	0.1342	0.1800	0.2005	0.1976	0.1901	0.1923	0.1611		0.0168	0.0193	0.0183	0.0164	0.0331
14	*Promuscidea unfasciativentris*	0.1832	0.1774	0.1209	0.1635	0.1683	0.1645	0.2071	0.2075	0.2057	0.2007	0.1913	0.1867	0.1256		0.0190	0.0177	0.0174	0.0340
15	*Chartocerus* sp1	0.1967	0.2012	0.1556	0.1800	0.1934	0.1887	0.2106	0.2433	0.2283	0.2261	0.2115	0.1976	0.1700	0.1629		0.0108	0.0154	0.0345
16	*Chartocerus* sp2	0.1838	0.1939	0.1470	0.1738	0.1812	0.1655	0.2102	0.2221	0.2116	0.2145	0.1953	0.2069	0.1576	0.1498	0.0642		0.0136	0.0334
17	*Chartocerus* sp3	0.1805	0.1846	0.1439	0.1624	0.1613	0.1571	0.2159	0.1962	0.2089	0.2146	0.2031	0.1812	0.1308	0.1354	0.1177	0.0986		0.0306
18	*Allotropa phenacocca*	0.3262	0.3467	0.3104	0.3310	0.3091	0.3255	0.3755	0.3904	0.3849	0.3792	0.3887	0.3759	0.3653	0.3577	0.3800	0.3634	0.3266	

Table S6. Genetic distances between parasitoid species between COI sequences under K2P model

	Species	1	2	3	4	5	6	7	8	9	10	11	12	13	14	15	16	17	18
1	*Acerophagus* sp1		0.0138	0.0207	0.0191	0.0204	0.0174	0.0203	0.0187	0.0194	0.0210	0.0208	0.0207	0.0206	0.0204	0.0203	0.0187	0.0190	0.0268
2	*Acerophagus* sp2	0.0938		0.0226	0.0183	0.0207	0.0189	0.0209	0.0177	0.0200	0.0216	0.0216	0.0214	0.0205	0.0196	0.0213	0.0197	0.0199	0.0266
3	*Aenasius arizonensis*	0.2162	0.2290		0.0187	0.0184	0.0184	0.0208	0.0195	0.0218	0.0220	0.0226	0.0234	0.0206	0.0221	0.0223	0.0206	0.0210	0.0298
4	*Anagyrus jenniferae*	0.1813	0.1795	0.1835		0.0146	0.0140	0.0169	0.0138	0.0190	0.0198	0.0189	0.0199	0.0172	0.0184	0.0201	0.0179	0.0182	0.0274
5	*Anagyrus kamali*	0.2160	0.2091	0.1847	0.1290		0.0156	0.0176	0.0151	0.0198	0.0198	0.0203	0.0205	0.0190	0.0190	0.0202	0.0176	0.0186	0.0272
6	*Anagyrus tristis*	0.1719	0.1791	0.1829	0.1198	0.1518		0.0173	0.0131	0.0188	0.0189	0.0192	0.0192	0.0193	0.0184	0.0201	0.0167	0.0194	0.0273
7	*Cheiloneurus nankingensis*	0.2155	0.2036	0.2134	0.1532	0.1729	0.1625		0.0187	0.0184	0.0180	0.0168	0.0204	0.0194	0.0213	0.0222	0.0203	0.0199	0.0283
8	*Gyranusoidea indica*	0.1816	0.1651	0.1898	0.1173	0.1375	0.1108	0.1789		0.0173	0.0192	0.0199	0.0206	0.0182	0.0179	0.0182	0.0158	0.0180	0.0272
9	*Prochiloneurus javanicus*	0.1891	0.2045	0.2332	0.1917	0.1909	0.1703	0.1891	0.1491		0.0165	0.0153	0.0204	0.0186	0.0197	0.0186	0.0204	0.0211	0.0281
10	*Prochiloneurus testaceus*	0.2241	0.2406	0.2389	0.2065	0.2086	0.1958	0.1838	0.2091	0.1585		0.0153	0.0226	0.0185	0.0215	0.0206	0.0201	0.0210	0.0278
11	*Prochiloneurus stenopterus*	0.2051	0.2104	0.2281	0.1738	0.1922	0.1658	0.1453	0.1904	0.1241	0.1452		0.0195	0.0188	0.0211	0.0209	0.0213	0.0217	0.0282
12	*Marietta picta*	0.2145	0.2140	0.2457	0.1966	0.2127	0.1796	0.1965	0.2066	0.1933	0.2342	0.1736		0.0209	0.0199	0.0226	0.0200	0.0202	0.0294
13	*Myiocnema comperei*	0.1939	0.1991	0.2206	0.1814	0.1861	0.1909	0.2056	0.1578	0.1769	0.2038	0.1871	0.2117		0.0190	0.0193	0.0177	0.0180	0.0251
14	*Promuscidea unfasciativentris*	0.2062	0.1925	0.2332	0.1745	0.2014	0.1820	0.2176	0.1724	0.1966	0.2414	0.2097	0.1863	0.1954		0.0184	0.0180	0.0199	0.0269
15	*Chartocerus* sp1	0.2023	0.2222	0.2344	0.2071	0.2124	0.1970	0.2477	0.1570	0.1732	0.2151	0.2115	0.2358	0.1898	0.1656		0.0149	0.0148	0.0279
16	*Chartocerus* sp2	0.1942	0.1967	0.2149	0.1766	0.1700	0.1486	0.2081	0.1376	0.1849	0.2073	0.2054	0.1850	0.1581	0.1611	0.1143		0.0142	0.0260
17	*Chartocerus* sp3	0.1843	0.2011	0.2318	0.1817	0.1875	0.1862	0.2234	0.1772	0.2115	0.2148	0.2203	0.1971	0.1649	0.1970	0.1253	0.1115		0.0269
18	*Allotropa phenacocca*	0.2899	0.2952	0.3553	0.3210	0.3429	0.3026	0.3424	0.2939	0.3286	0.3384	0.3172	0.3470	0.2656	0.3135	0.3082	0.2763	0.2877	

Table S7. Genetic distance within parasitoid species under K2P model

	Species	K2P distance	
		COI	28S
1	*Acerophagus* sp1	0.00%±0.00%	0.00%±0.00%
2	*Acerophagus* sp2	0.14%±0.07%	0.17%±0.10%
3	*Aenasius arizonensis*	0.76%±0.23%	0.00%±0.00%
4	*Anagyrus jenniferae*	0.00%±0.00%	0.00%±0.00%
5	*Anagyrus kamali*	0.19%±0.13%	0.07%±0.07%
6	*Anagyrus tristis*	0.00%±0.00%	0.00%±0.00%
7	*Cheiloneurus nankingensis*	0.00%±0.00%	0.03%±0.03%
8	*Gyranusoidea indica*	0.00%±0.00%	0.00%±0.00%
9	*Prochiloneurus javanicus*	0.26%±0.11%	0.00%±0.00%
10	*Prochiloneurus testaceus*	2.33%±0.55%	0.00%±0.00%
11	*Prochiloneurus stenopterus*	0.45%±0.21%	0.00%±0.00%
12	*Marietta picta*	0.09%±0.09%	0.00%±0.00%
13	*Myiocnema comperei*	0.00%±0.00%	0.00%±0.00%
14	*Promuscidea unfasciativentris*	0.49%±0.15%	0.00%±0.00%
15	*Chartocerus* sp1	0.19%±0.17%	0.00%±0.00%
16	*Chartocerus* sp2	0.00%±0.00%	0.00%±0.00%
17	*Chartocerus* sp3	0.00%±0.00%	0.00%±0.00%
18	*Allotropa phenacocca*	1.07%±0.30%	0.02%±0.02%

Table S8 K2P pairwise distances (%) of the COI and 28S gene within different taxonomic levels of the investigated parasitoids.

category	COI			28S		
	minimum	mean	maximum	minimum	mean	maximum
intraspecific	0.00%	0.33%	2.33%	0.00%	0.02%	0.17%
intrageneric	0.00%	3.78%	8.73%	0.00%	1.50%	7.01%
intrafamily	1.07%	9.38%	14.85%	0.02%	7.60%	13.45%
interspecific	9.38%	20.30%	35.53%	3.25%	20.42%	39.04%
intergeneric	11.90%	21.62%	35.53%	12.09%	22.03%	39.04%
interfamily	18.11%	25.52%	33.68%	16.13%	25.98%	36.75%
